# 1366. Temporal changes in Demographics, Characteristics and Treatments for Patients enrolled in the Canadian Treatments for COVID-19 Trial

**DOI:** 10.1093/ofid/ofad500.1203

**Published:** 2023-11-27

**Authors:** Samiha Mohsen, Srinivas Murthy, Ruxandra Pinto, Robert Fowler

**Affiliations:** University of Toronto, Toronto, Ontario, Canada; Department of Pediatrics, Faculty of Medicine, University of British Columbia, Vancouver, British Columbia, Canada; University of Toronto, Toronto, Ontario, Canada; Sunnybrook Health Sciences Centre, ON, Canada

## Abstract

**Background:**

Over 4 million Canadians have been infected with COVID-19. The risk to the Canadian population has been dynamic over time, with changing demographics of patients at risk, differential uptake of vaccination, and potentially differential presentation, access, or acceptance of evolving treatments. Our study aims to evaluate the temporal change in patient characteristics, process of care and outcomes over the pandemic for patients with COVID-19 admitted to hospitals and enrolled in the Canadian Treatments for COVID-19 trial (CATCO).

**Methods:**

The study included all patients admitted to 52 participating Canadian hospitals with laboratory confirmed SARS-COV-2 infection and enrolled in the CATCO trial. Data was analyzed temporally over six periods of enrollment, corresponding to approximately every 242-294 patients (Figure 1). Patient characteristics and outcomes were summarized using descriptive statistics (i.e., median, proportions).

**Figure:**
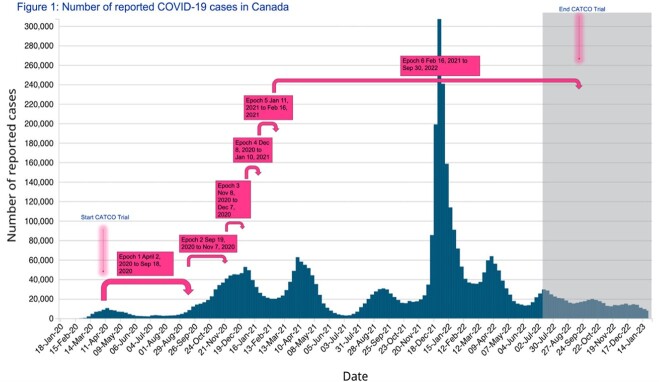

**Results:**

Mean age (63 years) and sex (30% female) among enrolled patients were similar across six pandmiec periods. Patient admission to ICU was most common at the beginning of the pandemic (period 1 n=75, 30.4%; period 2 n=67, 27.2%; approximately 20% in subsequent periods [p< 0.001]). The proportion of patients who identified as Black decreased from 10.2% to 3.1% between the first and sixth period, while the proportion of other minority groups remained stable (Figure 2.). Treatment with corticosteroids increased substantially after the first period (41.2% to over 90% in each subsequent period). Unadjusted in-hospital and 60-day mortality was similar over periods (p=0.501).

**Figure:**
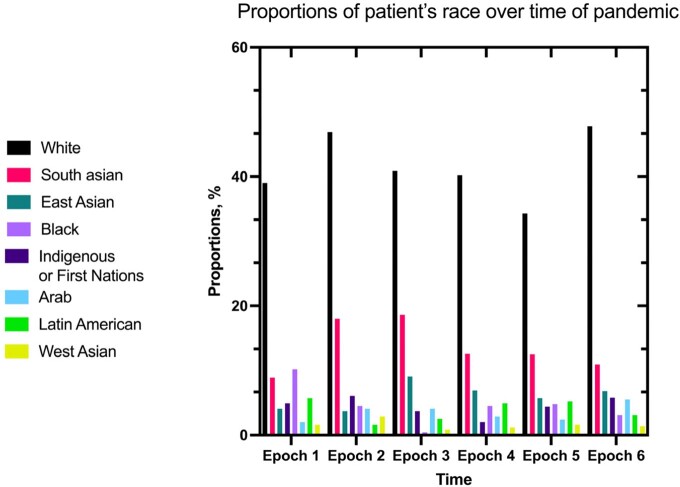

**Conclusion:**

There were changes in treatments and a decreasing proportion of enrolled patients admitted to ICU as enrollment in CATCO progressed. In contrast to epidemiological data that showed a change in demographics of hospitalized persons shifting from predominantly Caucasians to predominantly minority groups overtime, our study found that there was not increasing enrollment of minority groups over time. In a dynamic pandemic, it may be important to include the potential for temporal changes in patient characteristics, treatments, and support when investigating the effect of medications on clincial outcomes over time.

**Disclosures:**

**All Authors**: No reported disclosures

